# Characterization and Application of Statewide Emergency Medical Services Advanced Life Support Protocols

**DOI:** 10.7759/cureus.92053

**Published:** 2025-09-11

**Authors:** Priya Devanarayan, Rodney Sena, Daniel Johnson

**Affiliations:** 1 Department of Emergency Medicine, Penn State Health Milton S. Hershey Medical Center, Hershey, USA; 2 Ronald O. Pearlman Department of Emergency Medicine, NYU Langone Health, New York City, USA

**Keywords:** advanced life support, clinical protocol, decision making, emergency medical services, prehospital

## Abstract

Introduction: Many emergency medical services (EMS) systems in the United States utilize statewide protocols, but the number, type, and role of these systems in prehospital decision-making are largely unknown.

Study Objective: To characterize statewide protocols and determine their role in prehospital decision-making.

Methods: All states were queried for the presence of mandatory statewide Advanced Life Support (ALS)-level EMS protocols. Protocols were categorized as diagnosis-based, symptom-based, procedural, or non-clinical by two fellowship-trained EMS physicians. A data abstraction guide with category examples was included. Discrepancies were resolved by a third blinded EMS physician. The number and type of protocols were compiled, and simple statistics and chi-squared analysis were used to evaluate state-by-state variation.

Results: Nine states have mandatory statewide ALS protocols, totaling 804 individual protocols, including 672 (83.6%) clinical and 132 (16.4%) non-clinical protocols. Symptom-based protocols accounted for 59.5% of the clinical protocols, while 13.2% were diagnosis-based and 27.2% procedural. Per state, there was a median interquartile range (IQR) of 46 (14.5) symptom-based, 8 (8) diagnosis-based, and 17 (22.5) procedural protocols. There was significant variation in the type and number of protocols by state (p < 0.001).

Conclusion: Significant variation exists in statewide EMS protocols. While many are symptom-based, a notable portion are diagnosis-based, challenging the notion that paramedics cannot diagnose. These findings suggest a need for higher-level clinical decision-making in EMS. Further research is warranted at other EMS training levels and to inform EMS training programs.

## Introduction

Emergency medical services (EMS) systems in the United States operate with varying levels of direct and indirect medical oversight [1]. Standing orders, or protocols, dictate most of the care routinely provided by EMS clinicians. Requirements for direct oversight by a physician may either be written into protocols or provided as requested by the EMS clinician. Some states have universally applied statewide protocols adhered to by all EMS agencies. Others allow variation between services at the discretion of their physician medical directors. The breadth, type, and application of these protocols to the framework of prehospital decision-making are not well described [2].

As more complex care now takes place outside of the hospital by non-physicians, the role of the EMS “clinician” has garnered much support [3-5]. Historically, those who provided emergency medical care outside the hospital were described as “technicians.” Their role was to closely follow predetermined treatment protocols, with physician consultation frequently required [6-7]. Currently, there is a body of evidence suggesting that clinical decisions are being made independently by EMS clinicians in dynamic situations that are not always clearly represented in the protocols or treatment guidelines [8-9].

Research conducted with an aim to understand clinical decision-making in EMS has utilized predominantly qualitative methods to help determine how EMS providers make decisions and what information is utilized [2,3,7,9]. This study aims to characterize protocols from states that publish mandatory statewide protocols. This approach also aims to provide commentary on how protocols are likely incorporated into prehospital decision-making and the types of decisions expected to be made by EMS clinicians.

## Materials and methods

All states in the United States were queried for the presence of statewide EMS protocols at the advanced life support (ALS) level. This does not include states with mandatory protocols that can be edited at the discretion of the agency medical director. A categorization guide was created and provided to each data abstractor prior to data analysis and included the categories, criteria for inclusion into each category, and an example (Table 1). A Microsoft Excel spreadsheet of each protocol, by name, was provided to each data abstractor along with an electronic link to review the full text of the protocol, if desired. Two fellowship-trained EMS physicians were then asked to assign a category to each individual protocol. Discrepancies were adjudicated by a third equally qualified physician blinded to the research question. The number and type of each protocol were compiled, and summary statistics were performed in Microsoft Excel (Microsoft Corporation, Redmond, WA). A chi-squared analysis using SAS statistical software (SAS Institute, Cary, North Carolina, USA) was performed to determine if a variation of protocol type/number existed by state. A summary of this project was submitted to the Pennsylvania State University Institutional Review Board (IRB), which was determined not to meet the definition of human subject research and, therefore, was exempted from full IRB review and approval (Penn State University IRB STUDY 00022141).

**Table 1:** ALS protocol data abstraction guide provided to EMS fellowship-trained physicians detailing each protocol category and examples. ALS: advanced life support; DNR: do not resuscitate; EMS: emergency medical services

Category	Explanation	Example
Medical treatment (diagnosis-based)	A protocol that outlines the treatment or management of a particular condition or diagnosis.	ST-elevation myocardial infarction
Medical treatment (symptom-based)	A protocol that outlines the general approach to a chief complaint or symptom.	Chest pain
Procedural	A protocol that addresses a particular medical procedure or means/methods of administering a particular medication.	Needle thoracostomy; epinephrine auto-injector administration
Non-clinical	A protocol that addresses a particular standardized policy, scope of practice, or standard operating procedure.	Reporting elder or child abuse; charting standardization; out-of-hospital DNR; termination of resuscitation; transport destinations; use of air medical services; mass casualty incidents

## Results

Mandatory statewide ALS protocols exist in nine (18%) states: Delaware, Hawaii, Maine, Maryland, New Hampshire, New Jersey, Pennsylvania, Rhode Island, and West Virginia. Among these states, there are a total of 804 individual ALS protocols, including 672 (83.6%) clinical and 132 (16.4%) non-clinical protocols (Table 2). Symptom-based protocols accounted for 59.5% of the clinical protocols, while 13.2% were diagnosis-based and 27.2% procedural. Per state, there was a median interquartile range (IQR) of 46 (14.5) symptom-based, 8 diagnosis-based, and 17 (22.5) procedural protocols (Table 3) [8]. Significant variation in the number and type of protocols exists per state (p<0.001).

**Table 2:** Distribution of all ALS protocols across nine states with mandated protocols.

State	Protocol Type (n, %)				Total Number of Protocols
	Clinical			Non-clinical	
	Diagnosis-based	Complaint or Symptom-based	Procedural		
Delaware	8 (16.0%)	26 (52.0%)	5 (10.0%)	11 (22.0%)	50
Hawaii	8 (11.3%)	43 (60.6%)	13 (18.3%)	5 (7.0%)	69
Maine	7 (6.9%)	54 (53.5%)	17 (16.8%)	23 (22.8%)	101
Maryland	19 (20.4%)	52 (55.9%)	5 (5.4%)	16 (17.2%)	92
New Hampshire	12 (10.3%)	47 (40.5%)	26 (22.4%)	31 (26.7%)	116
New Jersey	5 (4.8%)	44 (42.3%)	33 (31.7%)	15 (14.4%)	97
Pennsylvania	7 (13.2%)	34 (64.2%)	9 (17.0%)	3 (5.7%)	53
Rhode Island	17 (12.0%)	54 (38.0%)	53 (37.3%)	18 (12.7%)	142
West Virginia	6 (7.1%)	46 (54.8%)	22 (26.2%)	10 (11.9%)	84

**Table 3:** Distribution of clinical ALS protocols across nine states with mandated protocols.

State	Clinical Protocol Type (n, %)			Total Number of Clinical Protocols
	Diagnosis-based	Complaint or Symptom-based	Procedural	
Delaware	8 (20.5%)	26 (66.7%)	5 (12.8%)	39
Hawaii	8 (12.5%)	43 (67.2%)	13 (20.3%)	64
Maine	7 (9.0%)	54 (69.2%)	17 (21.8%)	78
Maryland	19 (25.0%)	52 (68.4%)	5 (6.6%)	76
New Hampshire	12 (14.1%)	47 (55.3%)	26 (30.6%)	85
New Jersey	5 (6.1%)	44 (53.7%)	33 (40.2%)	82
Pennsylvania	7 (14.0%)	34 (68.0%)	9 (18.0%)	50
Rhode Island	17 (13.7%)	54 (43.5%)	53 (42.7%)	124
West Virginia	6 (8.1%)	46 (62.2%)	22 (29.7%)	74

## Discussion

Among the mandatory statewide ALS protocols in the United States, a significant portion were related to medical treatment, and variation exists between states. Most clinical medical treatment protocols were symptom-based, followed by procedural, and finally diagnosis-based. Each state also included non-clinical protocols related to general operations. This seems to suggest that the primary intent of statewide protocols is to guide the clinician through the evaluation of a particular chief complaint or presentation. While less prevalent, diagnosis-based protocols were ubiquitous among all states. Notably, the purpose of the study was not to determine the accuracy or consistency of this categorization among the investigators but rather to analyze the frequency of protocol type.

Medical treatment protocols can assist in several phases of the decision-making process. They can help the EMS clinician work through the approach to a particular chief complaint, such as atraumatic chest pain. They can also provide treatment guidelines if a working diagnosis is made. This study found that a smaller but not insignificant number of protocols addressed a particular diagnosis, such as ST-elevation myocardial infarction. This implies that EMS clinicians would be tasked with developing a framework to make this diagnosis, even in atypical situations. Similarly, while a significant portion of the protocols are procedural, the indications for such procedures must be clearly identified through the medical decision-making process.

This study suggests the formation of a working diagnosis may be a critical skill for EMS clinicians to apply diagnosis-based or procedural protocols. In a state such as Maryland, this represents nearly one-third of the total clinical protocols. While this process is studied extensively in medical and nursing education, its nuance is lacking in EMS curricula [10,11]. It has been shown that EMS clinicians rely heavily on experience and recollection of similar patient encounters, which can predispose the clinician to cognitive bias [12,13]. This information can guide education and training programs for EMS clinicians of the future. Education in clinical decision-making, and its pitfalls, is likely an important part of EMS curricula to better prepare EMS clinicians to make accurate and efficient medical decisions and avoid common cognitive biases.

The fact that significant variation exists between states suggests heterogeneity in the decision-making support provided by statewide protocols. States with higher proportions of symptom-based protocols may heavily support the diagnostic process, while others may emphasize treatment algorithms. Both approaches could prove beneficial. Diagnostic error is an area of particular risk in EMS medicine [14]. In addition, support in the form of diagnostic and treatment algorithms can allow the clinician to shift to a more methodical approach, which is less prone to cognitive bias [13]. Systems that favor diagnosis over symptom-based protocols need to ensure that clinicians have a strong understanding of the decision-making process in the out-of-hospital environment, including its limitations and pitfalls.

Limitations

In this study, two of the three data abstractors were members of the study team and not blinded. Additionally, as the categorization was based on the title and general contents of the protocol, there is a chance that some of the symptom-based protocols reference the diagnosis-based protocols and work synergistically. There is also a chance the protocols were miscategorized due to limited information provided. More research analyzing the intricacies of the protocols may be required to gain a greater understanding of this area. Finally, this study only analyzed ALS protocols and only those from nine states. While these are likely to contain the highest complexity decision-making, further studies are necessary to evaluate at other EMS training levels. There may also be state or regional variation that was not elucidated due to the inclusion criteria.

## Conclusions

Among states with mandatory statewide ALS protocols, variation in the type of protocol per state exists. Many protocols address medical treatment, while others are procedural or operational. Among the medical treatment protocols, a portion is diagnosis-based, which may suggest a higher level of decision-making among EMS clinicians. The process of formulating working diagnoses from undifferentiated complaints should be an area of focus of EMS education.
